# *Aeromonas hydrophila* ST251 and *Aeromonas dhakensis* are major emerging pathogens of striped catfish in Vietnam

**DOI:** 10.3389/fmicb.2022.1067235

**Published:** 2023-01-26

**Authors:** Kerry L. Bartie, Thao P. H. Ngô, Michaël Bekaert, Dang Thi Hoang Oanh, Rowena Hoare, Alexandra Adams, Andrew P. Desbois

**Affiliations:** ^1^Institute of Aquaculture, Faculty of Natural Sciences, University of Stirling, Stirling, United Kingdom; ^2^Aquacultural Biotechnology Division, Biotechnology Center of Ho Chi Minh City, Ho Chi Minh City, Vietnam; ^3^Department of Aquatic Pathology, Can Tho University, Can Tho, Vietnam

**Keywords:** antibiotic resistance, antimicrobial resistance (AMR), aquaculture, comparative genomics, hypervirulent *Aeromonas hydrophila* (vAh), motile *Aeromonas* septicaemia, pangasius, *Pangasianodon hypophthalmus*

## Abstract

**Introduction:**

Aeromonads are ubiquitous in aquatic environments and several species are opportunistic pathogens of fish. Disease losses caused by motile *Aeromonas* species, particularly *Aeromonas hydrophila*, can be challenging in intensive aquaculture, such as at striped catfish (*Pangasianodon hypophthalmus*) farms in Vietnam. Outbreaks require antibiotic treatments, but their application is undesirable due to risks posed by resistance. Vaccines are an attractive prophylactic and they must protect against the prevalent strains responsible for ongoing outbreaks.

**Methods:**

This present study aimed to characterize *A. hydrophila* strains associated with mortalities in striped catfish culture in the Mekong Delta by a polyphasic genotyping approach, with a view to developing more effective vaccines.

**Results:**

During 2013–2019, 345 presumptive *Aeromonas* spp. isolates were collected at farms in eight provinces. Repetitive element sequence-based PCR, multi-locus sequence typing and whole-genome sequencing revealed most of the suspected 202 *A. hydrophila* isolates to belong to ST656 (*n* = 151), which corresponds to the closely-related species *Aeromonas dhakensis*, with a lesser proportion belonging to ST251 (*n* = 51), a hypervirulent lineage (vAh) of *A. hydrophila* already causing concern in global aquaculture. The *A. dhakensis* ST656 and vAh ST251 isolates from outbreaks possessed unique gene sets compared to published *A. dhakensis* and vAh ST251 genomes, including antibiotic-resistance genes. The sharing of resistance determinants to sulphonamides (*sul1*) and trimethoprim (*dfrA1*) suggests similar selection pressures acting on *A. dhakensis* ST656 and vAh ST251 lineages. The earliest isolate (a vAh ST251 from 2013) lacked most resistance genes, suggesting relatively recent acquisition and selection, and this underscores the need to reduce antibiotics use where possible to prolong their effectiveness. A novel PCR assay was designed and validated to distinguish *A. dhakensis* and vAh ST251 strains.

**Discussion:**

This present study highlights for the first time *A. dhakensis*, a zoonotic species that can cause fatal human infection, to be an emerging pathogen in aquaculture in Vietnam, with widespread distribution in recent outbreaks of motile *Aeromonas* septicaemia in striped catfish. It also confirms vAh ST251 to have been present in the Mekong Delta since at least 2013. Appropriate isolates of *A. dhakensis* and vAh should be included in vaccines to prevent outbreaks and reduce the threat posed by antibiotic resistance.

## 1. Introduction

*Aeromonas* spp. are ubiquitous members of autochthonous communities in aquatic environments, while some species are opportunistic pathogens of animals and humans (Janda and Abbott, 2010; Batra et al., 2016; Pessoa et al., 2019; Fernández-Bravo and Figueras, 2020) meaning they can present a One Health challenge (Lamy et al., 2021). Infections caused by *Aeromonas* spp. can be particularly challenging at aquaculture sites where aquatic animals are farmed intensively, as these provide ideal conditions for a disease outbreak. Vietnam is a major aquaculture producer with several species dominating output, including the striped catfish *Pangasianodon hypophthalmus* (Sauvage, 1878), which is exported around the world as pangasius (Phuong and Oanh, 2010). Striped catfish production is concentrated in the Mekong Delta region where these fish are cultured primarily in freshwater ponds and the sector provides significant support to the regional economy with exports worth >USD 1.7 bn, thereby securing livelihoods and employment (Phuong and Oanh, 2010; Nguyen and Jolly, 2020; Hasan and Shipton, 2021). However, bacterial disease outbreaks can disrupt production, with the most important pathogens being *Edwardsiella ictaluri*, which causes bacillary necrosis of pangasius (BNP), and motile species of *Aeromonas*, particularly *A. hydrophila*, which are responsible for motile *Aeromonas* septicaemia (MAS), also known as haemorrhagic or red spot disease (Phu et al., 2016; Hoa et al., 2021). In striped catfish, MAS manifests clinically with hemorrhage, abscess, ulcers, ascitic fluid, and anemia, and outbreaks result typically in high rates of mortality (Anyanwu et al., 2015; Phu et al., 2016).

An injectable vaccine is available commercially in Vietnam to protect against BNP and MAS, and it contains an inactivated isolate of *E. ictaluri* and two biotypes of *A. hydrophila* (ALPHA JECT^®^ Panga 2, PHARMAQ; Tung et al., 2014); however, uptake by farmers is far from universal with cost cited as a barrier because specialized teams and equipment are needed to administer the vaccine (Adams, 2019; Kayansamruaj et al., 2020). Consequently, treatment with antibiotics is needed when outbreaks occur (Rico et al., 2013; Ström et al., 2019), but this is undesirable due to risks associated with the selection and enrichment of antibiotic-resistant strains in the environment and in people and animals exposed to these agents (Mo et al., 2015; Phu et al., 2016; Brunton et al., 2019; Hoa et al., 2021). Therefore, the implementation of preventative measures to counter infectious diseases, including easy to administer and less costly vaccines, is a priority, with oral and immersion-based vaccines showing promise and in advanced development (Mzula et al., 2019; Ngo et al., 2022). Nevertheless, to ensure greatest efficacy, it is critical that newly developed vaccines protect against the most prevalent disease-causing lineages in circulation, thus underlining a need for regular surveillance.

Due to long standing misidentification issues and the high heterogeneity present within the *Aeromonas* genus (Pessoa et al., 2019), the strains affecting striped catfish deserve special attention. Several approaches can be applied to differentiate strains of *Aeromonas*, although even speciation within this genus can be a challenge due to traditional phenotypic markers lacking sufficient discrimination (Pessoa et al., 2019; Fernández-Bravo and Figueras, 2020). Indeed, the molecular epidemiology of MAS outbreaks affecting striped catfish in the Mekong Delta is not well described, and many studies have relied solely on traditional biochemical tests to identify presumed *Aeromonas* spp. (Nguyen et al., 2014), with identification of *A. hydrophila* reliant on single genetic markers based on amplification of the aerolysin gene (Pollard et al., 1990; Hoa et al., 2021). More recently, genetic methods of characterisation for *Aeromonas* spp. based on housekeeping gene phylogeny and multi-locus sequence typing (MLST) have proven popular and these can provide more reliable species placement and strain resolution (Martino et al., 2011; Navarro and Martínez-Murcia, 2018). Still, instances of ambiguous species attribution have been documented based on MLST (de Melo et al., 2019), with whole genome sequencing (WGS) providing the most definitive differentiation of species and strains (Bayliss et al., 2017). Such genomic studies have revealed the presence of a hypervirulent lineage (vAh) of *A. hydrophila* [sequence type (ST) 251] that is prompting serious concern in global aquaculture due to its role in causing MAS outbreaks in farmed channel catfish and carp in the USA and China, respectively (Pang et al., 2015; Rasmussen-Ivey et al., 2016; Awan et al., 2018), whilst vAh ST251 isolates have been detected recently in two provinces farming striped catfish in Vietnam (Ngo et al., 2022). Application of a comprehensive suite of strain typing methods to isolates associated with MAS outbreaks in Vietnam would allow for the identification of the predominant strains in circulation for inclusion into more effective vaccines, whilst providing insight into the most suitable methods for future surveillance to ensure their continued effectiveness.

Therefore, the aim of the present study was to characterize the circulating *A. hydrophila* strains associated with mortality losses in striped catfish culture in the Mekong Delta, Vietnam by a polyphasic genotyping approach, with a view to applying this knowledge to the development of new vaccines offering greater protection against this pathogen.

## 2. Materials and methods

### 2.1. Reference and type strains

Five *A. hydrophila* isolates of varied host origin were used for reference purposes and these isolates originated from Thailand (isolate F2D20 from *Rana rugulosa*), Bangladesh (isolate T4 from *Labeo rohita* and isolate B2/12 from an unknown host), India (isolate VDS from *Ictalurus punctatus*), and USA (isolate AL09-71 from *I. punctatus*). A further reference isolate, *Aeromonas veronii* biovar *sobria* LMG 13067 (originating from an unspecified frog host in the USA), and 12 type strains of *Aeromonas* spp. were also included (Supplementary Table 1).

### 2.2. Sampling sites and primary isolation of *Aeromonas* spp. field isolates

*P. hypophthalmus* exhibiting classical signs of MAS, including reddened fins and external and/or internal hemorrhage, were sampled from disease outbreaks in the Mekong Delta region (An Giang, Ben Tre, Can Tho, Dong Thap, Hậu Giang, Tien Giang, and Vinh Long provinces) and Dong Nai province during 2013–2019. For each sampling occasion, at least two representative field isolates were included that had been derived from separate farms located in the same geographic area. Additionally, nine farms from five regions were sampled more extensively between 2018 and 2019, where multiple fish, internal organs and isolates per culture plate were collected. After disinfecting the fish surface with 70% ethanol in water, the internal organs (liver, spleen, and head kidney) were dissected out aseptically, streaked on to Rimler-Shotts agar (RS; Himedia, India) or *Aeromonas* medium (supplemented with ampicillin; Thermo Fisher Scientific, UK) and incubated overnight at 28°C. Smooth, convex, round, and yellow (or dark green on *Aeromonas* medium) single colonies presumed to be *Aeromonas* spp. were sub-cultured in tryptone soya broth (TSB; Himedia) at 28°C for 16–20 h at 240 rpm. All isolates, samples and locations are listed in Supplementary Table 1. Cultures were cryopreserved at –80°C in TSB medium supplemented with 20% glycerol (Merck, USA).

### 2.3. Diagnostic PCR to confirm colonies to be *A. hydrophila*

Genomic DNA was extracted from the culture pellet of each isolate using the GeneJET Genomic DNA Purification kit (Thermo Scientific, USA). PCR was performed against the *16S rRNA* gene using the primers of Trakhna et al. (2009), and this assay yields an amplicon of 103 bp in the presence of genomic DNA from *A. hydrophila*. This *16S rRNA* PCR assay was chosen to identify *A. hydrophila* instead of the more traditional aerolysin gene PCR (Pollard et al., 1990), as initial evaluations found equivalent PCR outcomes with reference isolates within the *Aeromonas* genus whilst avoiding the presence of non-specific artifact products that appeared for a small proportion of aerolysin PCR-negative samples collected from the field (e.g., isolate TN120 from An Giang and isolates TN103 and TN117 from Dong Thap; data not shown). Each PCR contained 10 ng genomic DNA in a 10 μL total volume containing 1 × MyTaq HS PCR master mix (Meridian Bioscience, UK) and 0.2 μM of each primer. The amplification conditions were as follows: initial denaturation at 95°C for 3 min, followed by 30 cycles of 95°C for 15 s, 55°C for 15 s, and 72°C for 10 s. PCR amplicons were electrophoresed through 1% agarose gel (Meridian Bioscience, UK) containing 0.1 mg/mL ethidium bromide in 0.5 × TAE (20 mM Tris, 10 mM acetic acid, and 0.5 mM EDTA). The gel was observed under UV light using the InGenius gel imaging system (Syngene, UK) for the presence of the expected band.

### 2.4. Repetitive element sequence-based PCR (rep-PCR)

Each field isolate was genotyped using a rep-PCR based on the single (*GTG*)_5_ repetitive primer, as described previously (Bartie et al., 2012). Field isolates negative by *16S rRNA* PCR were included to see whether lineages of non-*A. hydrophila* species associated strongly with MAS outbreaks (Supplementary Table 2). Five microlitres of each PCR reaction was separated on a 1.5% UltraPure Agarose-1000 gel (Invitrogen, Thermo Fisher Scientific) in chilled 0.5 × TAE buffer. A GeneRuler 100 bp Plus DNA Ladder (Thermo Fisher Scientific) was used to aid DNA profile comparisons. Following electrophoresis, the gels were stained for 30 min in 1 mg/mL ethidium bromide and de-stained in Milli-Q water (Merck Millipore, UK) for 1 h. DNA profiles resulting from the rep-PCR were visualized and gel images captured using the InGenius system (Syngene, UK). Numerical analysis of the DNA fingerprints was performed using Gel Compar II software (Applied Maths NV, Belgium). Dendrograms were constructed using the unweighted pair group method with arithmetic mean (UPGMA) and Pearson similarity coefficient. Clusters of similar banding profiles (similarity of at least 95%) were defined as an equivalent rep-PCR type where the banding patterns were considered indistinguishable by manual inspection of the respective rep-PCR gel profiles.

### 2.5. Phenotypic characterisation of suspected *A. hydrophila* isolates

A subset of 12 field isolates positive by *16S rRNA* PCR, representative of the main groups by rep-PCR and collected from different provinces and sampling times, were analyzed by API 20E biochemical profiling (bioMérieux, USA) and oxidase test (Oxoid) to further support the species identification (Supplementary Table 2). *A. hydrophila* subsp. *hydrophila* ATCC 7966^T^ was included for comparison.

### 2.6. Next-generation sequencing multi-locus sequence typing (ngsMLST)

A subset of 132 field isolates were selected for ngsMLST profiling such that they represented the diversity of *A. hydrophila* diagnostic PCR outcomes, rep-PCR profiles and sample sites. Four *A. hydrophila* reference isolates of varied host origin and six type strains of *Aeromonas* spp. were included as control material for ngsMLST library preparation (Supplementary Table 2). A total of nine MLST loci and the full-length *16S rRNA* gene (Weisburg et al., 1991) were amplified by PCR, barcoded, and sequenced using the high-throughput sequencing HiSeq platform (Illumina, USA) in order to assess the genetic variation within the field isolate collection. Six loci were included (*gyrB, groL, glt*A, *met*G, *ppsA, recA*) based on the MLST scheme of Martino et al. (2011) with three additional markers (*rpoD, dnaX, dnaJ*) selected for their ability to inform phylogeny within the *Aeromonas* genus (Nhung et al., 2007; Martinez-Murcia et al., 2011). Primer sequences of the MLST loci and full-length *16S rRNA* gene are listed in Supplementary Table 3.

### 2.7. ngsMLST library preparation

The ten loci of interest were amplified individually in a 5-μL PCR containing 1 × MyTaq HS mix (Meridian Bioscience, UK), 0.2 μM of each primer pair and 2.5 ng genomic DNA template. The touchdown cycling protocol consisted of an initial denaturation at 95°C for 3 min; then 10 cycles of 95°C for 15 s, annealing at 65°C (with this temperature decreasing by 1°C each cycle), and extension at 72°C for 30 s; followed by eight PCR cycles performed at an annealing temperature of 55°C. The full-length *16S rRNA* gene PCR was conducted in the same PCR master mix conditions with an initial 3 min denaturation step at 95°C; 15 cycles of 95°C for 15 s, 45°C for 30 s, and 72°C for 45 s; and a final extension at 72°C for 5 min.

Amplicons were visualized on a 1.5% TAE agarose gel and the PCR products from each isolate combined to normalize total DNA template according to band intensity and size. Samples were purified by AxyPrep Mag PCR bead clean-up kit (Axygen Biosciences, USA) at a bead ratio of 0.6 × and diluted to 0.2 ng/μL according to Qubit dsDNA HS quantification (Invitrogen; Thermo Fisher Scientific). Libraries were constructed in a miniaturized volume modified from an existing Illumina Nextera XT Library Preparation protocol (Illumina, USA), starting with 0.1 ng DNA template in a 2.5 μL tagmentation mixture and 5 μL barcoding PCR. Then libraries were cleaned up by AxyPrep magnetic beads (Axygen Biosciences, USA) at a 1:1 ratio. Finally, quantified libraries (Qubit dsDNA HS) were normalized by abundance of DNA, divided into four pools, and submitted for sequencing on a NovaSeq 6000 sequencing platform (Illumina, USA) at PE250 reads (Novogene, UK).

### 2.8. ngsMLST data analysis

Mass-parallel molecular typing data (ngsMLST sequences) were filtered for quality (QC > 20), length (150 nt), and absence of primers/adaptors and complexity (entropy > 15) using fastp (Chen et al., 2018). Gene sequence assemblies and typing were performed using SRST2 v0.2.0 (Inouye et al., 2014) and PubMLST (Jolley and Maiden, 2012). The resulting sequences of nine MLST loci and the full-length *16S rRNA* gene with average coverage above 400 × were concatenated and aligned using GramAlign v3.0 (Russell, 2014). A phylogenetic tree was generated by PhyML v3.3.20200621 (Guindon et al., 2010) and this included concatenated ngsMLST sequences derived from published genomes of two reference isolates (vAh ST251 strain AL09-71 and *A. veronii* biovar *sobria* LMG 13067) and 12 *Aeromonas* spp. type strains (Supplementary Table 2).

### 2.9. Whole-genome sequencing

Fourteen isolates positive by the *A. hydrophila 16S rRNA* diagnostic PCR from the predominant rep-PCR types, and the reference isolate *A. hydrophila* T4 that originated from *L. rohita* in Bangladesh (Poobalane et al., 2010), were selected for WGS. Genomic DNA samples were submitted for DNA library preparation and microbial WGS (Novogene, UK) on the NovaSeq 6000 sequencing platform (Illumina, USA) at PE150 read length.

### 2.10. Genome analysis

Reads from the 15 sequencing libraries were used separately during the assembly process and reads were filtered as described in Section 2.8. Raw data were assembled using Spades v3.14.0 (Bankevich et al., 2012) and the Unicycler-pipeline v0.4.8 (Wick et al., 2017). The initial *de novo* output was re-aligned against both *A. dhakensis* CIP 107500^*T*^ (assembly GCF_000820305.1), a closely related species frequently misidentified as *A. hydrophila*, and *A. hydrophila* subsp. *hydrophila* ATCC 7966^T^ (assembly GCF_000014805.1), using CONTIGuator v2.7.5 (Galardini et al., 2011) to order the contigs when continuity was incomplete. Finally, Pilon v1.23 (Walker et al., 2014) was used for polishing and correcting sequencing errors and to recover closed circular plasmid sequences. Subsequently, all genomes were annotated by DFAST v1.2.4 (Tanizawa et al., 2018) and plasmids identified using blastN v2.11.0 (Nowicki et al., 2018) against the National Center for Biotechnology Information (NCBI) plasmid database [2021–12–01]. Furthermore, genome sequences (including complete sequences, draft assemblies, and raw reads) of 116 publicly available *A. dhakensis* and *A. hydrophila* strains, and four other *Aeromonas* spp. to act as outgroups, were downloaded from the European Bioinformatics Institute (Supplementary Table 4). Unassembled samples were assembled according to the same process as the newly sequenced isolates.

### 2.11. Species affiliation by average nucleotide identity (ANI)

To support species assignments, FastANI v1.33 (Jain et al., 2018) was applied to the genome sequences to calculate an ANI index against several type strains, specifically *A. dhakensis* CECT 7289^T^ and CIP 107500^T^ (assemblies GCF_000819705.1 and GCF_000820305.1, respectively), and *A. hydrophila* subsp. *hydrophila* ATCC 7966^T^ (GCF_000014805.1). An ANI value of more than 0.96 was selected as the species threshold (Ciufo et al., 2018).

### 2.12. MLST typing and ST-eBurst

MLST types were determined for each genome with mlst v2.19.0 (Seemann, 2022) and PubMLST (Jolley and Maiden, 2012). The Phyloviz v2.0a (Nascimento et al., 2017) tool was used to run the goeBURST nLV algorithm and visualize trees based on the probable patterns of evolutionary descent between allelic profiles.

### 2.13. Core and accessory genomes

PIRATE v1.0.4 (Bayliss et al., 2019) was used to create a detailed pan-genome and to classify the core and accessory genomes of all available *A. dhakensis* and *A. hydrophila* isolates. Analysis of pan-genome outputs was performed using R v4.0.0 (R Core Team, 2022). Data on clustering and presence/absence, and trees from PIRATE, were visualized using panX release bb56978 (Ding et al., 2018) and phandango v1.3.0 (Hadfield et al., 2018).

### 2.14. Screening for antibiotic resistance genes

Screening for the presence of antibiotic resistance genes was conducted for the genomes with ABRicate v1.0.0 (Seemann, 2020), using multiple databases [2021–12–01]: NCBI (Feldgarden et al., 2020) and PlasmidFinder (Carattoli et al., 2014).

### 2.15. Design and validation of primers to distinguish the main groups of strains

Core genes of the two most prevalent groups of strains observed in this present study were used to design pairs of discriminating primers, with conserved regions within core genes used to identify sites for the anchoring primers. Then, whole genomes were scanned to confirm the specificity of the primer sets using ecoPrimer v0.5 (Riaz et al., 2011). Candidate PCR primer pairs (targeting sequences in *yjcS* and *intA_5* genes) were evaluated against a panel of field isolates and reference and type strains to confirm their specificity (Supplementary Table 2). Each PCR contained 5 ng genomic DNA in a 10 μL total volume containing MyTaq HS mix (Meridian Bioscience, UK) and 0.2 μM of each primer. Initial specificity testing was conducted using the following amplification conditions: denaturation at 95°C for 3 min; followed by 30 cycles of 95°C for 15 s, 52°C for 15 s, and 72°C for 10 s. PCR amplicons were separated through a 1% agarose gel (Meridian Bioscience, UK) containing 0.1 mg/mL ethidium bromide and visualized. A gradient PCR between 52°C and 62°C was conducted to optimize the PCR conditions and confirm the differential amplification of DNA from isolates representing the different groups of strains.

## 3. Results

### 3.1. Collection of field isolates

In total, 345 presumptive *Aeromonas* spp. colonies were recovered on isolation agar plates from *P. hypophthalmus* individuals exhibiting classical signs of MAS. Of these presumed *A. hydrophila* isolates, 58.6 (202/345) yielded the expected 103-bp amplicon in the PCR of Trakhna et al. (2009) that targets specifically the *16S rRNA* gene of *A. hydrophila*, thus indicating the presence of this species (Supplementary Table 2). Of the nine farm sites sampled between 2018 and 2019, PCR-positive isolates suspected to be *A. hydrophila* were detected at seven sites, including co-isolation with PCR-negative colonies at six of these sites, indicative of the presence of other *Aeromonas* spp. Notably, of the 40 fish from which more than one isolate was recovered from the internal organs, in eight cases PCR-positive and negative colonies were isolated, indicating the presence of more than one *Aeromonas* spp. (e.g., Site 3, Fish 1; Supplementary Tables 1, 2).

A summary of the analyses performed for isolates included in this present study can be found in Supplementary Figure 1.

### 3.2. Repetitive PCR (rep-PCR) genotyping

Analysis of the 345 field isolates by rep-PCR revealed complex DNA profiles consisting of 12–14 fragments, with each ranging from ca. 300–3,000 bp (Supplementary Figure 2). Of the 202 isolates positive by *16S rRNA* PCR and thus suspected to be *A. hydrophila*, two distinct profiles were dominant: these were confirmed by manual inspection and numerical analysis and termed rep-PCR types A and B (Supplementary Table 2). Rep-PCR types A and B were widely distributed across the eight provinces sampled (Supplementary Figure 2), with rep-PCR type A being most prevalent (151/202; 74.8%) among the suspected *A. hydrophila* isolates, especially in samples collected more recently. Of note, from one fish (Site 3, Fish 3) was isolated three PCR-positive colonies, where two isolates clustered within rep-PCR type A whilst the third was rep-PCR type B (Supplementary Tables 1, 2).

More variable rep-PCR profiles were observed for the colonies isolated on Aeromonas selective medium that were negative by the *16S rRNA* PCR (Supplementary Table 2, Supplementary Figure 3), and these presumed *Aeromonas* spp. isolates formed relatively diffuse clusters (Supplementary Figure 2) compared to the tight clusters corresponding to the two main rep-PCR types, A and B of suspected *A. hydrophila* isolates that tested positive by *16S rRNA* PCR.

### 3.3. Phenotypic characterisation of suspected *A. hydrophila* isolates

Biochemical characterisation of *A. hydrophila* subsp. *hydrophila* ATCC 7966^T^ and 12 field isolates, suspected to be *A. hydrophila* by *16S rRNA* PCR and representative of rep-PCR types A and B, revealed identical API-20E biochemical profiles (704126) matching *A. hydrophila*, according to BacDive [https://bacdive.dsmz.de/api-test-finder].

### 3.4. ngsMLST phylogeny

**Figure 1:**
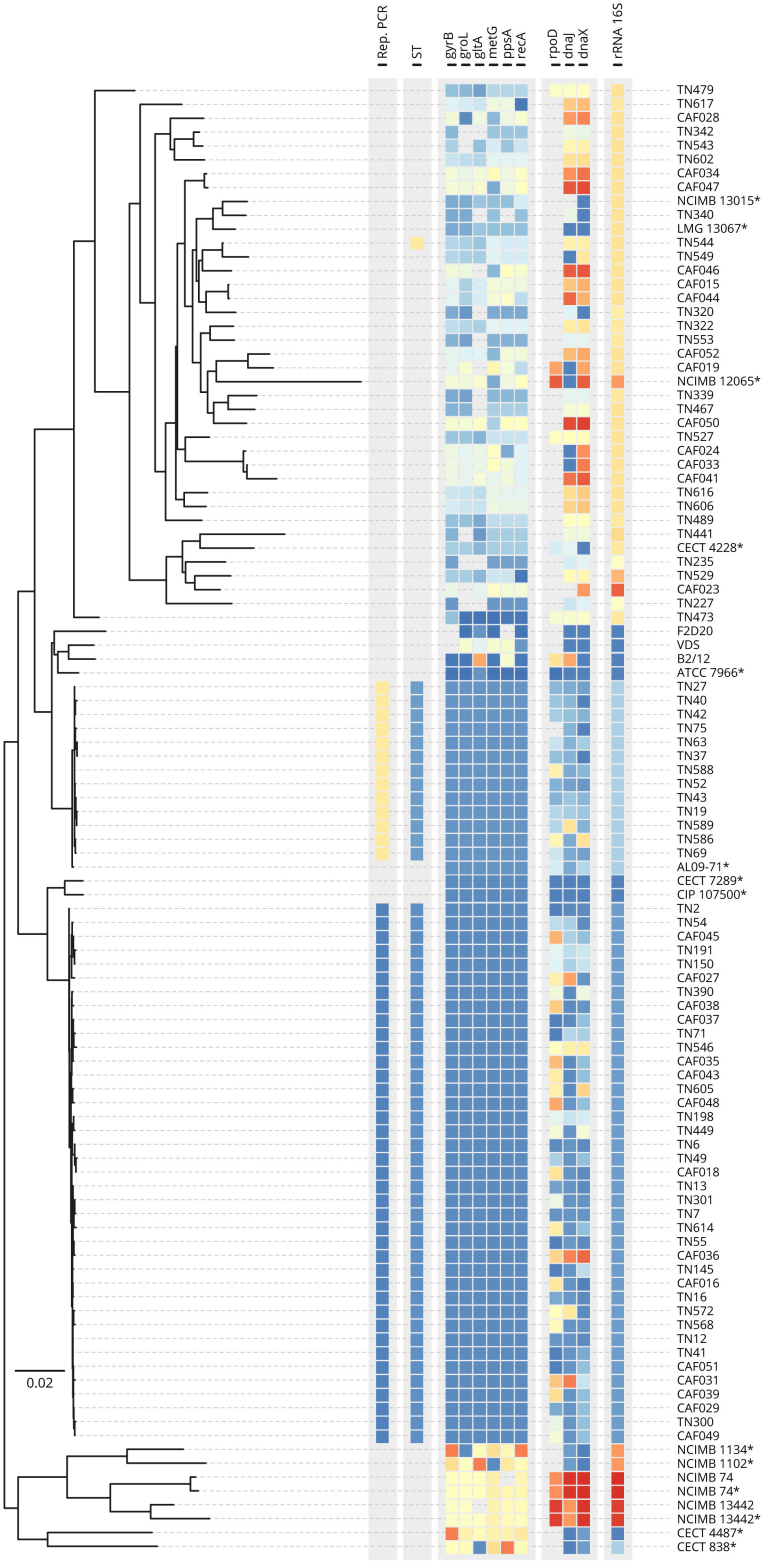
Multilocus phylogenetic analysis by sequence comparison of concatenated housekeeping genes at 10 loci for 87 representative field isolates of *Aeromonas* from striped catfish, five reference isolates, and 12 type strains. rep-PCR type, sequence type (ST), and alleles derived from ngsMLST analysis of 10 genes including *16S rRNA* are color coded. *Gene sequences (when available) were retrieved from the genome assembly at NCBI.

To provide greater confidence into the identities and relatedness of the field isolates, ngsMLST was performed whereby fragments (between 477 and 1,496 bp) at each of the 10 loci were amplified for 132 representative field isolates, four *A. hydrophila* reference isolates from varied hosts, and six *Aeromonas* spp. type strains. These isolates were used as template for ngsMLST library preparation and were selected for diversity of sampling location, and the outcomes of *16S rRNA* PCR and rep-PCR assays (Supplementary Table 2). After applying the selected depth threshold, a phylogenetic tree was generated for 92 libraries (87 field isolates, three reference isolates, and two type strains) based on the concatenated sequence (ca. 6,910 bp in length) from each isolate (Figure 1). Amongst the field isolates suspected to be *A. hydrophila* (i.e., positive by *16S rRNA* PCR), two major clusters formed according to multilocus phylogenetic analysis (MLPA). Application of the PubMLST scheme and allelic profiling of the six housekeeping genes (*gyrB, groL, gltA, metG, ppsA*, and *recA*) permitted the assignment of sequence types (STs) to each of these two main clusters, namely ST656 (*n* = 39) and ST251 (*n* = 13), which corresponded to rep-PCR types A and B respectively. ST656 is associated with *A. dhakensis* (Jolley et al., 2010), a species closely related to *A. hydrophila* (Beaz-Hidalgo and Figueras, 2013), while ST251 is a hypervirulent lineage of *A. hydrophila* (vAh). In contrast, the PCR-negative *Aeromonas* spp. isolates clustered more variably and distinctly from the main *A. dhakensis* and vAh ST251 clades and formed a diffuse cluster that affiliated most closely to published loci of *A. veronii* NCIMB 13015^T^ (*n* = 26) with a minor sub-cluster related to *A. jandaei* CECT 4228 and *A. sobria* NCIMB 12065^T^ (*n* = 9; Figure 1). The remaining three field isolates from *P. hypophthalmus* presented as outlier groups according to MLPA. The majority of the allelic profiles of these *Aeromonas* spp. isolates did not match any ST in the PubMLST *Aeromonas* database (Supplementary Table 2).

### 3.5. Genome assemblies and ANI

**Figure 2:**
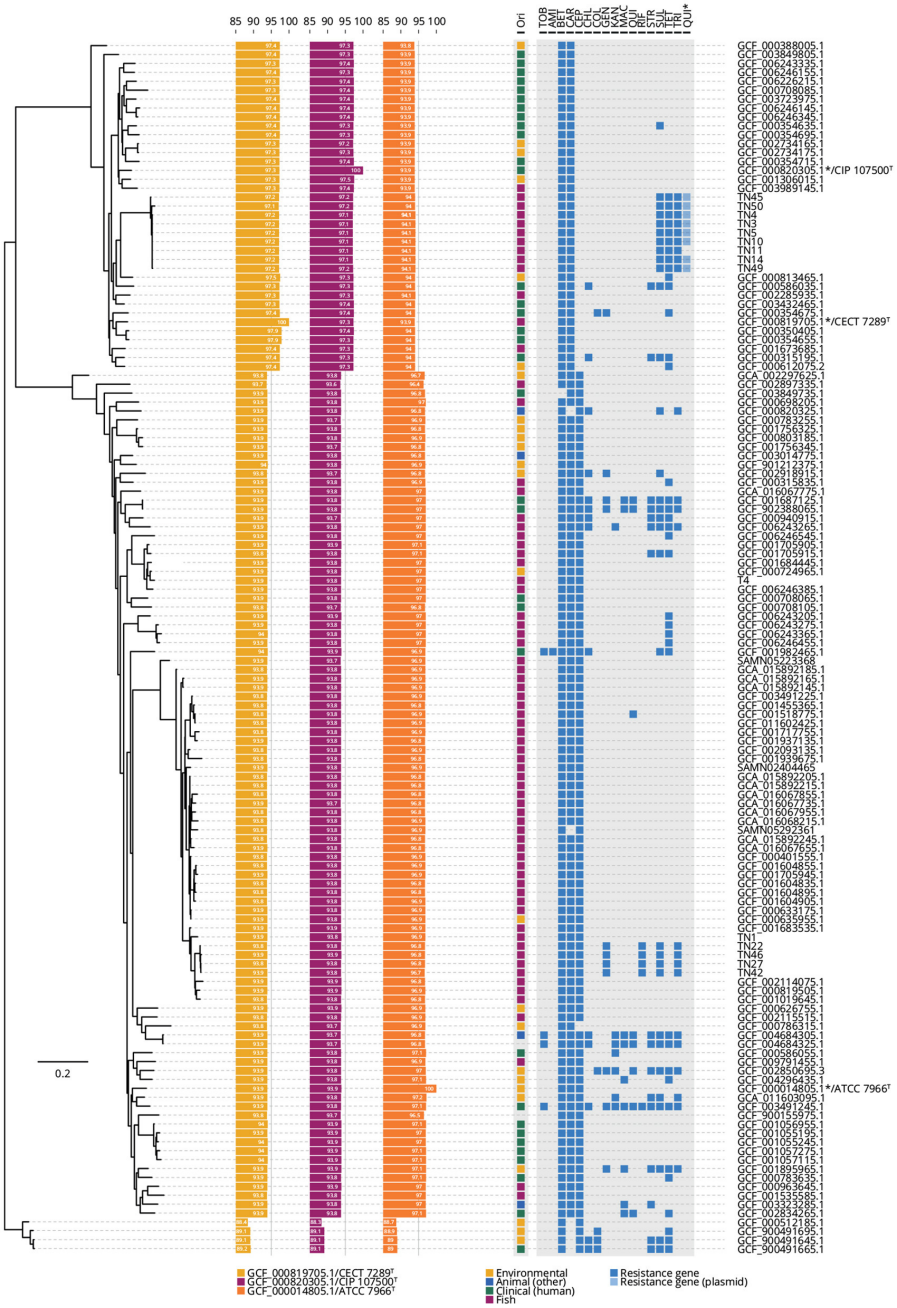
Phylogenetic tree of 14 field isolates, one reference isolate, and 116 published *A. hydrophila* and *A. dhakensis* genomes. Four *Aeromonas* spp. genomes are included in the analysis as an outlier group. Tree is based on the pan-genome analysis. Average nucleotide identity (ANI) values were calculated compared to the three labeled type strains (*A. dhakensis* CECT 7289^*T*^ assembly GCF_000819705.1; *A. dhakensis* CIP 107500^*T*^ assembly GCF_000820305.1; and *A. hydrophila* subsp. *hydrophila* ATCC 7966^*T*^ assembly GCF_000014805.1). The origin (Ori) of each isolate and the presence/absence of 17 antibiotic resistance genes in the pan-genome analysis is provided. *The quinoline resistance gene was found on a plasmid and plasmid sequences were available only for the genomes sequenced in this present study. Full list of the abbreviations and detailed gene names is provided in Supplementary Table 5.

Genome sequencing generated a mean of 12 million short-reads for each of 14 representative field isolates and the *A. hydrophila* T4 reference strain (Table 1). The genomes ranged between 4.82 and 4.98 Mb, with GC ratios ranging from 60.9 to 61.4%; several closed plasmids were detected consistently (Table 2). All genomes were (re-)annotated for gene location consistency using DFAST (Supplementary Table 4), aligned against selected type strains to determine ANI (Supplementary Table 5), and re-classified (Figure 2). Field isolates fell within two clades that associated with either *A. dhakensis* or the clonal vAh ST251 lineage within *A. hydrophila*, respectively. Moreover, the ANI calculations supported the existence of these two predominant species amongst the isolates collected from *P. hypophthalmus* (Figure 2, Supplementary Table 5). ANI values of 97.1–97.2% were estimated for nine of the genomes (all rep-PCR type A) against the two *A. dhakensis* type strains (CECT 7289^T^ and CIP 107500^T^). Similarly, the five vAh ST251 genomes (all rep-PCR type B) associated most closely with the *A. hydrophila* subsp. *hydrophila* ATCC 7966^T^ type strain (mean ANI value of 96.8%). The reference strain, *A. hydrophila* T4, was also placed within the major *A. hydrophila* clade (ANI values of 97.0% compared to *A. hydrophila* subsp. *hydrophila* ATCC 7966^T^), although separately from the ST251 lineage.

**Table 1:** Field isolate genomes sequenced in this present study.

**Isolate**	**Year**	**Country**	**Province**	**Host**	**Rep-PCR type**	**Read number**	**Run Acc**.
T4	1994	Bangladesh	–	*L. rohita*	*n.d*.	15,048,804	ERR4911987
TN1	2013	Vietnam	AG	*P. hypophthalmus*	B	18,305,234	ERR4911995
TN3	2013	Vietnam	AG	*P. hypophthalmus*	A	14,958,648	ERR4911948
TN4	2014	Vietnam	AG	*P. hypophthalmus*	A	14,771,162	ERR4911656
TN5	2013	Vietnam	AG	*P. hypophthalmus*	A	6,820,380	ERR4911952
TN10	2014	Vietnam	AG	*P. hypophthalmus*	A	7,456,818	ERR4911957
TN11	2014	Vietnam	AG	*P. hypophthalmus*	A	18,000,280	ERR4911967
TN14	2013	Vietnam	AG	*P. hypophthalmus*	A	8,281,592	ERR4911973
TN22	2014	Vietnam	CT	*P. hypophthalmus*	B	16,909,614	ERR4912005
TN27	2014	Vietnam	DT	*P. hypophthalmus*	B	7,555,950	ERR4912016
TN42	2015	Vietnam	BT	*P. hypophthalmus*	B	14,792,042	ERR4912885
TN45	2018	Vietnam	TG	*P. hypophthalmus*	A	10,315,308	ERR4911633
TN46	2015	Vietnam	DN	*P. hypophthalmus*	B	8,067,104	ERR4912011
TN49	2015	Vietnam	AG	*P. hypophthalmus*	A	7,221,844	ERR4911978
TN50	2015	Vietnam	AG	*P. hypophthalmus*	A	8,292,598	ERR4911638

For each isolate the year sampled, country of origin and province (AG, An Giang; CT, Can Tho; DT, Dong Thap; DN, Dong Nai; BT, Ben Tre; TG, Tien Giang), host, rep-PCR genotype (rep-PCR type A of *A. dhakensis* ST656 and rep-PCR type B of vAh ST251), total reads, and data accession number (Run Acc.) is provided. *n.d*., not determined.

**Table 2:** Genome assembly summary of 15 field isolate genomes.

**Isolate**	**Species**	**MLST type**	**Chromosome size (bp)**	**a**	**b**	**c**	**d**	**e**	**f**	**g**	**h**	**Assembly acc**.
T4	*A. hydrophila*	u3	4,891,336									GCA_905132965
TN1	*A. hydrophila*	vAh ST251	4,957,116							•	•	GCA_905132985
TN3	*A. dhakensis*	ST656	4,850,229	•	•	•	•					GCA_905132935
TN4	*A. dhakensis*	ST656	4,850,407	•	•	•	•					GCA_905132895
TN5	*A. dhakensis*	ST656	4,850,228	•	•	•	•					GCA_905132915
TN10	*A. dhakensis*	ST656	4,850,407	•	•	•	•					GCA_905132905
TN11	*A. dhakensis*	ST656	4,850,231	•	•	•	•					GCA_905132845
TN14	*A. dhakensis*	ST656	4,847,439	•	°	•	°					GCA_905132925
TN22	*A. hydrophila*	vAh ST251	4,977,638					•	•			GCA_905132975
TN27	*A. hydrophila*	vAh ST251	4,977,339					°	•			GCA_905132955
TN42	*A. hydrophila*	vAh ST251	4,978,631					▴	•			GCA_905132945
TN45	*A. dhakensis*	ST656	4,820,690	•	•	•	•					GCA_905132775
TN46	*A. hydrophila*	vAh ST251	4,977,942					•	•			GCA_905132995
TN49	*A. dhakensis*	ST656	4,850,028	•	•	•	•					GCA_905132875
TN50	*A. dhakensis*	ST656	4,847,436	•	•	•	•					GCA_905132865

For each isolate the species assignment, multi-locus sequence type, chromosome size, presence of plasmids and assembly accession number is provided. •, circular/complete plasmid sequence identified; °, partial plasmid sequence found; ▴, present with extra insertion (5,640 bp). a, p6.4-Qnr (6,386 bp); b, p6.3-RelE (6,276 bp); c, p6.2-StbE (6,162 bp); d, p4.7-HicB (4,678 bp); e, p5.0-YhdJ (5,092 bp); f, p3.6-parDE (3,663 bp); g, p6.2-ParE (6,214 bp); h, p4.1-Rec (4,140 bp).

### 3.6. Genome-based MLST and ST-eBurst analysis

**Figure 3:**
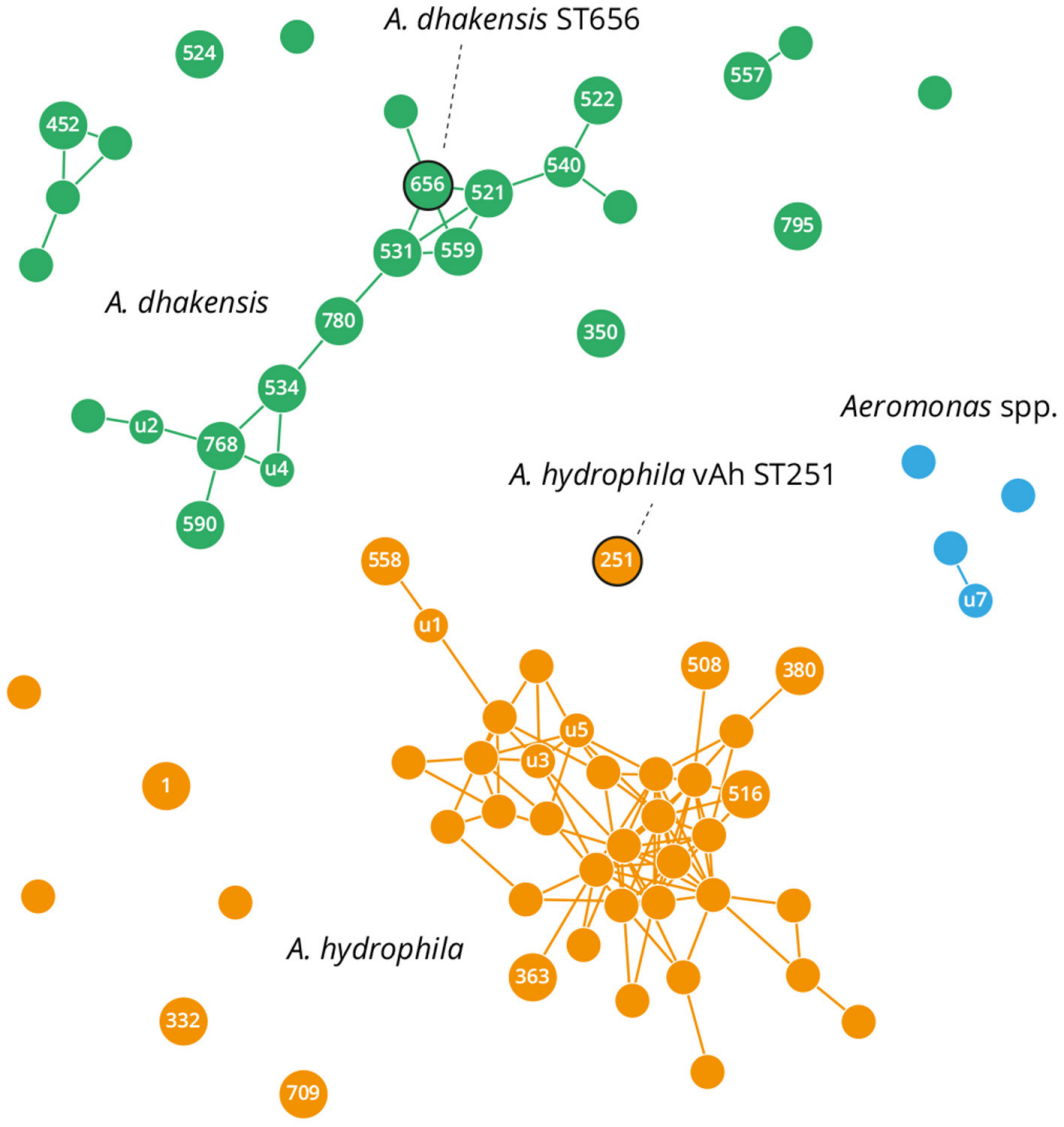
eBurst network based on multilocus sequence typing (MLST) patterns of publicly available *A. hydrophila* and *A. dhakensis* genomes. Circles denote a distinct MLST: small and unlabelled if undefined and unique; small and labeled if undefined but identified multiple times (“u”); and larger and labeled if present in the PubMLST database. Links show single allele variants. Sequence types (STs) containing field isolates are outlined in black and these correspond to *A. hydrophila* vAh ST251 and *A. dhakensis* ST656.

Genomes generated in this present study and all those publicly available for *A. dhakensis* and *A. hydrophila* were analyzed by MLST for the *Aeromonas* genus to identify STs and perform network analysis. Most of the genomes had profiles corresponding to a known ST, although 64 out of 135 analyzed genomes presented with novel gene sequences and MLST associations (denoted by “u” in Figure 3 and Supplementary Table 5). Network eBurst analysis based on sequence distance (Figure 3) revealed high diversity within and between the *A. dhakensis* and *A. hydrophila* species, thus supporting their separation into two species. For both species, strain STs were distributed typically as part of a network of single locus variants or less commonly as unique doublets or singletons (Figure 3). The *A. dhakensis* ST656 isolates associated with *P. hypophthalmus* were located within the main clonal complex of *A. dhakensis* strains; conversely, *A. hydrophila* ST251 isolated from *P. hypophthalmus* presented as a singleton.

### 3.7. Core and accessory genomes

**Figure 4:**
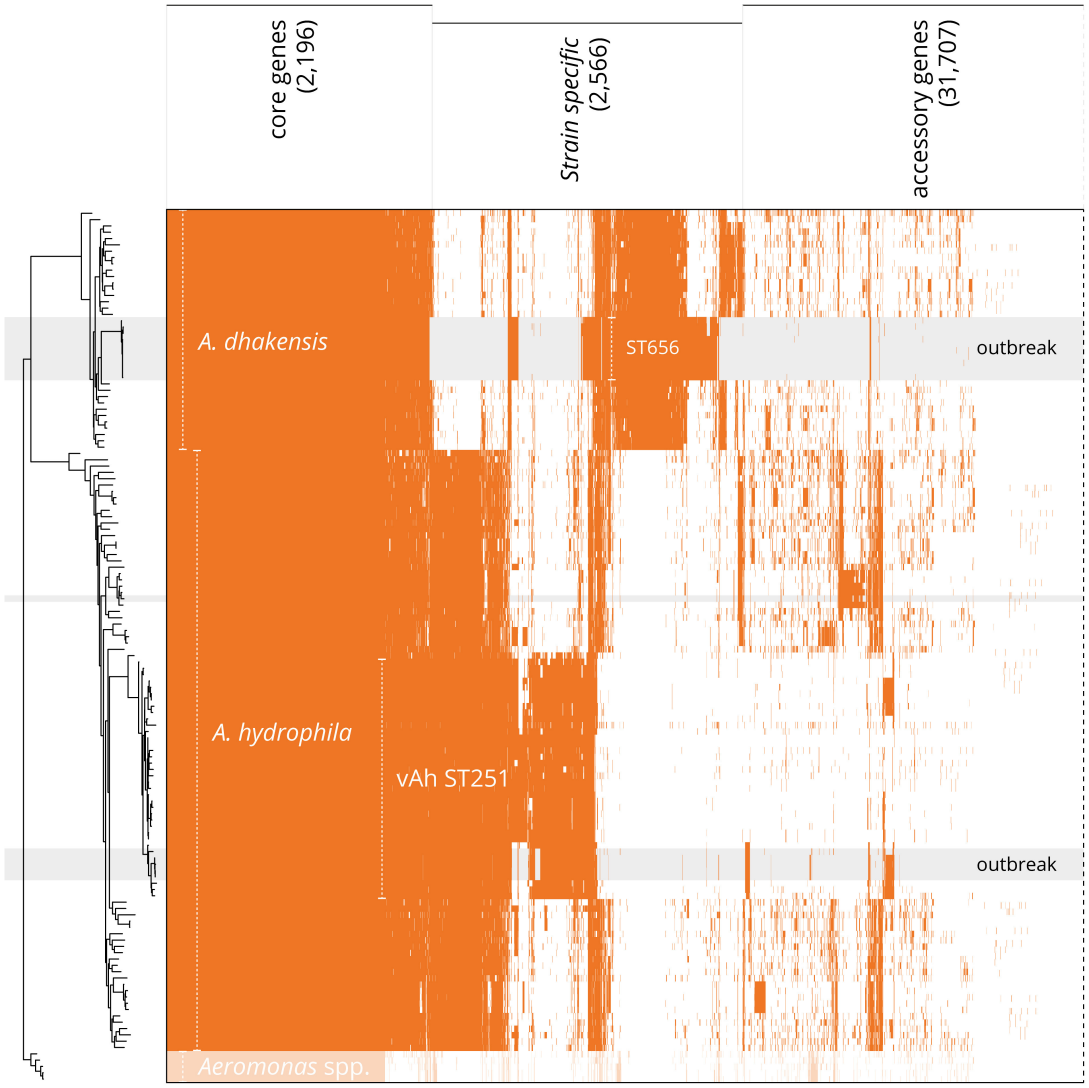
Distribution of protein-encoding genes in the pan-genome of *A. hydrophila* and *A. dhakensis*. Tree is based on pan-genome analysis, calculated by PIRATE using the accessory genes. Gray background indicates isolates sequenced *de novo* in this present study, with field isolates labeled “outbreak”. Shared protein-encoding genes constitute the core genome (95% presence; 2,517 genes). *A. dhakensis* has species-specific genes (698 genes) whilst the outbreak strains (ST656, from this present study) share a cluster of clone-specific genes (additional 769 genes). Similarly, *A. hydrophila* possesses 439 unique genes (95% presence), whilst the vAh ST251 strains exhibit a specific gene cluster distinct from all other specimens (additional 425 genes). A 4-way Venn diagram of the separate pan-genomes for *A. dhakensis, A. dhakensis* ST656, *A. hydrophila* and *A. hydrophila* vAh ST251 is available in Supplementary Figure 4.

Core (*n* = 2,517) and accessory genes (*n* = 33,952) were inferred to create a detailed pan-genome and to classify core and accessory genomes using all available *A. dhakensis* and *A. hydrophila* genomes (threshold of 95% presence in available genomes; Figure 4). When analyzing *A. dhakensis, A. dhakensis* ST656, *A. hydrophila* and *A. hydrophila* vAh ST251 pan-genomes separately, there were 2,196 shared core genes identified between the groups (threshold of 95% presence in available genomes for each group; Supplementary Figure 4). A characteristic pattern of genes within the core genomes distinguished the two closely related species of *A. dhakensis* (*n* = 1,505 unique genes not found in *A. hydrophila*) and *A. hydrophila* (*n* = 923 unique genes not found in *A. dhakensis*). Strain-specific regions associated with the *A. dhakensis* ST656 (*n* = 769 genes) and vAh ST251 (*n* = 425 genes) genotypes were detected when compared to all published *A. dhakensis* and *A. hydrophila* genomes, respectively (Supplementary Figure 4).

### 3.8. Antibiotic resistance genes

Screening for antibiotic resistance genes was conducted for each of the 135 *A. dhakensis* and *A. hydrophila* available genomes, including four *Aeromonas* spp. genomes as outliers (Figure 2, Supplementary Table 5). Interestingly, the genomes of the field isolates possessed sets of genes implicated in a multi-drug resistance trait and in patterns not detected in closely-related *A. dhakensis* and vAh ST251 counterparts (Figure 2, Supplementary Table 5). The nine field isolates of *A. dhakensis* ST656 each included resistance determinants to sulphonamides (*sul1*), trimethoprim (*dfrA1*), tetracycline (*tetA*), and a plasmid-mediated quinolone resistance gene (*qnrS2*). Of note, the sulphonamide (*sul1*) and trimethoprim (*dfrA1*) resistance genes were detected in both *A. dhakensis* ST656 and vAh ST251 outbreak genomes. Four of the vAh ST251 field isolates originating from multiple provinces in 2014 and 2015 also contained additional genetic elements conferring reduced susceptibility to gentamicin [*aac(6*′*)-Ib4*] and rifamycin (*arr-2*). Intriguingly, the earliest vAh ST251 isolate (TN1 collected in 2013) lacked most of these additional antibiotic-resistance determinants (Figure 2).

Gene determinants encoding reduced susceptibility to β-lactams and carbapenems were common in all *A. dhakensis* and *A. hydrophila* genomes, including in field isolates. The β-lactamase gene, bla_*AQU*_, was found uniquely in *A. dhakensis*, including the ST656 outbreak strains isolated from striped catfish, while the chromosomally mediated Class D OXA β-lactamase genes, bla_*OXA*−726_ or related bla_*OXA*−724_, were common to genomes of both species. Sequences with homology to CphA-type carbapenemases, belonging to metallo-β-lactamases subclass B2, were also shared amongst the two species, with the cphA3 subtype associated with vAh ST251, including in the five genomes of the vAh ST251 field isolates sequenced in this present study. In contrast, a predicted cephalosporin-resistant phenotype was limited to *A. hydrophila*, including in the vAh ST251 field isolates (*cepH*) and in the *A. hydrophila* T4 reference genome (*cepS*). Markers for reduced susceptibility to chloramphenicol, aminoglycosides, macrolides and quinolones were present but distributed more sporadically across the entirety of genomes examined.

### 3.9. Design and validation of primers to distinguish *A. dhakensis* and vAh ST251

Two primer sets based on the core genes in the *A. dhakensis* and vAh ST251 genomes were evaluated for their ability to distinguish these two key strain groups (Supplementary Table 6). To confirm the specificity of each set of primers, the two pairs were screened in an initial evaluation against a panel of 14 *Aeromonas* isolates that included field isolates of *A. dhakensis* ST656 (*n* = 2) and vAh ST251 (*n* = 2), non-vAh *A. hydrophila* reference strains (*n* = 2), *A. hydrophila* subsp. *hydrophila* ATCC 7966^T^, the *A. dhakensis* type strains (*n* = 2), and type strains of other *Aeromonas* spp. (*n* = 5). The PCR assay designed to target *yjcS*, which encodes a metallo-β-hydrolase in the *A. dhakensis* genome, yielded the predicted 223-bp amplicon at 62°C only for the DNA samples derived from the two *A. dhakensis* isolates collected from striped catfish in this present study (TN5 and TN49) and the two *A. dhakensis* type strains (CIP 107500^T^ and CECT 7289^T^). Similarly, a PCR assay targeting the *intA_5* integrase gene in the vAh ST251 genome yielded a 283-bp amplicon at the optimum 58°C annealing temperature for only the two vAh ST251 field isolates collected herein (TN1 and TN27). No amplification in either assay was observed for any non-target isolates (Supplementary Figure 5).

## 4. Discussion

This present study aimed to characterize the predominant strains of *A. hydrophila* causing MAS in striped catfish in the Mekong Delta, Vietnam to inform the selection of isolates for inclusion into new vaccine formulations. A polyphasic approach was taken to provide insight into the most appropriate methods for discriminating the strains sufficiently. Following selective culture of MAS-affected fish tissues on agar, biochemical profiling, species-specific PCR, rep-PCR, ngsMLST, and WGS, the main strains responsible for recent MAS outbreaks were determined to be clonal lineages of two emerging pathogens, specifically a hypervirulent clone of *A. hydrophila* (vAh ST251) and a predominant single clone of the closely related species *A. dhakensis* (ST656).

Isolates of *A. dhakensis* were most prevalent in the samples collected from striped catfish, with this species representing 151/202 of the field isolates testing positive by the *16S rRNA* PCR of Trakhna et al. (2009). This present study is the first to report *A. dhakensis* to be a major pathogen affecting the striped catfish sector in the Mekong Delta, Vietnam. *A. dhakensis* isolates were collected from all eight provinces during 2013-2019, and these isolates clustered within the rep-PCR type A group and belonged to ST656. The detection and clonal nature of *A. dhakensis* was unexpected because *A. hydrophila* is often cited to be the most important pathogen responsible for MAS in striped catfish in Vietnam (Ngo et al., 2022). However, given that *A. dhakensis* (Aravena-Román et al., 2011) and *A. hydrophila* (Beaz-Hidalgo et al., 2013) are frequently misidentified due to overlapping phenotypic traits, shifting classification schemes and a lack of reliable discriminatory tools (Awan et al., 2018; da Silva Filho et al., 2021), it is highly likely that *A. dhakensis* is an under-reported cause of mortality in aquatic animals, with disease outbreaks attributed instead to *A. hydrophila* (Aravena-Román et al., 2011). Previously, *A. dhakensis* (formerly *Aeromonas aquariorum*) has been reported in disease events of ornamental fish in Portugal (Martinez-Murcia et al., 2008) and Sri Lanka (Jagoda et al., 2014), and in eels in Spain (Esteve et al., 2012) and China (Guo et al., 2016). Moreover, *A. dhakensis* was the predominant species isolated from freshwater fish with MAS symptoms in Malaysia (Azzam-Sayuti et al., 2021), and this species was also implicated in disease outbreaks in farmed tilapia in Mexico (Soto-Rodriguez et al., 2013).

The remaining 51/202 field isolates that tested positive by the Trakhna et al. (2009) PCR grouped together as rep-PCR type B and were identified as vAh ST251. Isolates of this hypervirulent clone were found in the earliest samples analyzed, which confirms its presence in the striped catfish sector in the Mekong Delta since at least 2013 (TN1 was isolated in 2013; AG-2013-AG1 in Ngo et al., 2022). Furthermore, vAh ST251 isolates showed widespread geographical and temporal distribution through to 2019 when the most recent samples were cultured (Supplementary Table 1), demonstrating the persistence of this clone within the industry and supporting a major role for *A. hydrophila* in the etiology of MAS in striped catfish (Stratev and Odeyemi, 2017). Lineages of vAh ST251 have been implicated in significant MAS outbreaks worldwide including those affecting the catfish industry in the USA and carp production in China (Pang et al., 2015; Rasmussen-Ivey et al., 2016), and further sampling may reveal this clone to be responsible for disease outbreaks elsewhere. The restricted singleton presentation of vAh ST251 is unusual given the high strain diversity typically encountered in *A. hydrophila*. Further, the global dissemination of the vAh ST251 epidemic strain is limited to fish hosts thus far, and the pan-genomic analysis presented herein supports the existence of vAh-specific regions within core and accessory genomes that could explain the hypervirulence and host specificity phenotype (Pang et al., 2015; Rasmussen-Ivey et al., 2016; da Silva Filho et al., 2021). Indeed, gene clusters unique to the vAh ST251 lineage have been described previously in relation to prophages, O-antigen biosynthesis and fucose and myo-inositol metabolism (Hossain et al., 2013; Rasmussen-Ivey et al., 2016; Ngo et al., 2022).

This present study showed that only rep-PCR, MLST, and WGS had sufficient discriminatory power to distinguish the two main groups of isolates effectively, specifically vAh ST251 and *A. dhakensis*. Indeed, the vAh ST251 and *A. dhakensis* isolates were indistinguishable by colony appearance on RS agar, API20E biochemical profiles and the *16S rRNA* PCR (Supplementary Table 2). The inability of the Trakhna et al. (2009) primers to discriminate *A. dhakensis* from *A. hydrophila* is not entirely unexpected given the close genetic relatedness of these species (Awan et al., 2018). Nevertheless, that *A. dhakensis* ST656 returned an identical API20E profile to vAh ST251 (*i.e*., 704126) was unexpected because the inability of *A. dhakensis* to ferment L-arabinose (API20E profile, 7047125) has been applied to discriminate these species previously (Esteve et al., 2012). Still, the high strain diversity within the *Aeromonas* genus has resulted in isolates with atypical phenotypes being encountered, such as *A. dhakensis* 1P11S3 from striped catfish in Malaysia that utilized myo-inositol similar to vAh ST251 (Azzam-Sayuti et al., 2022). The inability of *A. dhakensis* to assimilate urocanic acid or to produce acid from L-fucose are other phenotypic markers that have been applied to distinguish *A. hydrophila* and *A. dhakensis* (Huys et al., 2002), but the observations in this present study further underscore the need for more effective discriminatory genotyping tools to differentiate *Aeromonas* species. Rep-PCR with a single repetitive primer such as (*GTG*)_5_ has long been exploited in bacterial strain typing due to the relative simplicity, low cost, and discriminative power of the assay to subspecies level (Ishii and Sadowsky, 2009), and the continued relevance of this genotyping methodology was demonstrated. Although rep-PCR performed well to describe the population structure of strains associated with the MAS outbreaks, MLST provides a more objective approach to genotyping and a scheme has been established for the *Aeromonas* genus (Martino et al., 2011; Du et al., 2021). MLST allows the assignment of definitive genotypes for reliable comparisons to previous studies, but the approach can be relatively costly through reliance on Sanger sequencing technology. To counter this shortcoming, in this present study a ngsMLST sequencing protocol was devised to create multiplexed miniaturized libraries from the PCR amplicons of each isolate at each MLST locus, followed by high-throughput sequencing as applied in yeast (Chen et al., 2015). This method allows for cost effective, large-scale strain characterization compared to more laborious traditional sequencing methods, and offers a highly discriminative approach for future surveillance efforts of bacterial pathogens, including those associated with MAS outbreaks as demonstrated in this present study. Further to this, to enable a rapid and inexpensive method to discriminate *A. dhakensis* from *A. hydrophila* and vAh ST251, a simple endpoint PCR was designed based on the core genomes. The primers were validated experimentally to confirm their specificity against a panel of strains that included field isolates, non-vAh isolates, and type strains of *A. dhakensis, A. hydrophila* subsp. *hydrophila* and several other *Aeromonas* spp. However, it is unclear whether these primers can differentiate between other strains of *A. dhakensis*, vAh ST251 of varied origin or *Aeromonas* species, and further work is required to confirm this. Even so, this *yjcS* PCR assay represents a novel tool that could be applied to improve detection of *A. dhakensis* and avoid misidentification as *A. hydrophila*, particularly for MAS outbreaks in striped catfish in the Mekong delta. Similar assays to the *intA_5* PCR used to identify vAh ST251 in this present study have been reported to target specific sequences unique to the strains from the United States (Griffin et al., 2013), as well as vAh lineages from outbreaks in Asia (Rasmussen-Ivey et al., 2016).

WGS provided the greatest discriminatory power and phylogenetic insight, and sequencing of 14 genomes representative of the two main clusters of striped catfish isolates confirmed definitively the respective species affiliations, their clonal nature and separation into two distinct lineages. The genomes for the *A. dhakensis* ST656 isolates generated in this present study are the first to be reported from striped catfish and are a welcome supplement to the relatively few genomes available for this species, compared to *A. hydrophila*. However, *A. dhakensis* is attracting increasing attention as an emerging pathogen with zoonotic potential, especially in Asia, where this species has been associated with serious infections and fatalities in humans (Huys et al., 2002; Wu et al., 2012; Chen et al., 2016; Khor et al., 2018; Kitagawa et al., 2019; Lau et al., 2020; Sun et al., 2021). Interestingly, though ST656 has been recovered exclusively so far from fish hosts, this clone was placed within a larger clonal complex of *A. dhakensis* strains originating from human clinical cases (Figure 3), meaning outbreaks of disease due to *A. dhakensis* in fish hosts present a theoretical risk to susceptible individuals interacting with infected stocks. Of note, a fatal case of necrotising fasciitis involving *A. dhakensis* was recorded in Australia after exposure to pond water (Melo-Bolivar et al., 2019). Pan-genomic analyses of the WGS data revealed genomic regions unique to *A. dhakensis* and a relatively large abundance of genes associated distinctively with the ST656 genotype, which could have a role in disease progression and host specificity (Supplementary Figure 4). Additional genomes for *A. dhakensis* and *A. hydrophila* and their analysis will enhance our understanding of zoonotic potential and the success of epidemic strains.

The genomic analyses confirmed the presence of antimicrobial resistance determinants in the field isolates of both species, which can reduce therapeutic treatment options and underlines the need for greater uptake of control measures by striped catfish farmers such as vaccines (Dien et al., 2022). The inherent reduced susceptibility of *Aeromonas* isolates to β-lactams is well recognized (Awan et al., 2018), but the genomes of the isolates collected from the striped catfish revealed the co-occurrence of resistance genes to sulphonamides, trimethoprim, tetracycline and quinolines in *A. dhakensis*, and to sulphonamides, trimethoprim, gentamicin, and rifamycin in vAh ST251. Of particular concern, the *qnrS2* gene that confers resistance to quinoline was detected on a 6.4 kb plasmid in *A. dhakensis*, which indicates a propensity for likely transmission between strains, as shown before for *Aeromonas* spp. isolates in this study arena (Nguyen et al., 2014). Notably, *A. hydrophila* T4 (isolated in 1994 from an Asian carp in Bangladesh) and the earliest vAh ST251 field isolate in this present study (TN1 sampled from a diseased pangasius catfish in 2013; Ngo et al., 2022) did not possess most of the additional antimicrobial resistance genes, in stark contrast with the more recent vAh ST251 genomes, thereby suggesting relatively recent acquisition and/or selection. The emergence of the multi-resistant strains has likely occurred in response to selective pressure exerted by exposure to antibiotics applied in the Mekong Delta region (Phu et al., 2016; Truong et al., 2019; Dang et al., 2021). Still, antibiotic susceptibility testing is needed to verify the reduced susceptibility phenotypes of the field isolates. Amongst the *Aeromonas* genomes, the frequent presence of resistance elements to agents reserved as treatments of last resort for Gram-negative infections in humans is of concern, particularly genes encoding resistance against carbapenems (*cphA*). Likewise, the frequent carriage of the chromosomal class C β-lactamase bla_*AQU*_ in *A. dhakensis*, including in the genomes of field isolates from striped catfish, has reduced therapeutic options in cases of septicaemia in humans (Wu et al., 2013), and *A. dhakensis* is postulated to be a reservoir of β-lactamase-encoding genes Yi et al. (2014).

Amongst the field isolates that tested negative by the *16S rRNA* PCR, high strain diversity was detected by rep-PCR and ngsMLST both within and between sampling sites (Supplementary Figure 2, Figure 1). Thus, the molecular epidemiology of *Aeromonas* strains causing MAS in striped catfish appears to be complex and this fits with a more opportunistic and sporadic population structure for these other species. Similar to the varied rep-PCR profiles encountered, the extensive strain diversity of other *Aeromonas* species relative to the single clonal lineages of *A. dhakensis* and vAh ST251 was indicated by less well-defined clusters in the MLPA of the concatenated housekeeping gene sequences and inability to assign isolates to an existing ST. The species identification for these *Aeromonas* spp. isolates was less certain, with closest homology to members of the *A. veronii* complex noted in the main MLPA cluster of suspected *Aeromonas* spp. isolates, and a minor group related to *A. jandaei* and *A. sobria* (Figure 1). This is consistent with other studies that have implicated species other than *A. hydrophila* in MAS outbreaks, including *A. veronii* in channel catfish in Vietnam (Hoai et al., 2019) and carp in China (Ran et al., 2018), and *A. veronii* and *A. jandaei* in tilapia in Thailand (Dong et al., 2017; Sakulworakan et al., 2021). This present study relied on opportunistic sampling and a structured epidemiological approach is necessary to understand the relative contributions of *A. hydrophila* including vAh ST251, *A. dhakensis* and other *Aeromonas* species and strains to MAS outbreaks in striped catfish in Vietnam. Moreover, isolation of more than one *Aeromonas* sp. from the fish sampled in this present study indicates that co-infections may be common in striped catfish, as has been reported for carp (Ran et al., 2018). In this present study, co-isolation of more than one *Aeromonas* sp. isolate was detected in 9/40 fish from which multiple isolates were collected, and this included a single fish (Site 3, Fish 3) from which both *A. dhakensis* ST656 and vAh ST251 were isolated (Supplementary Tables 1, 2). Furthermore, the presence of *Aeromonas* spp. with non-aeromonads in clinical infections is also not unusual (Fernández-Bravo and Figueras, 2020), with both *A. hydrophila* and *E. ictaluri* co-isolated from individual fish at nurseries in Vietnam (Hoa et al., 2021).

In conclusion, two epidemic lineages of *Aeromonas* were associated with recent outbreaks of MAS in striped catfish in the Mekong Delta of Vietnam, specifically *A. dhakensis* ST656 and vAh ST251. An endpoint PCR to differentiate these strains was designed and validated. The most appropriate and practical methods for continued surveillance of the strains underlying MAS outbreaks include rep-PCR and MLST, where a new approach relying on next-generation sequencing was developed (ngsMLST). Representative isolates of these two key pathogens can be used to develop improved vaccine formulations, including novel vaccines for mucosal delivery, and this forms the focus of a forthcoming study. Such vaccines delivered in feed or by bathing should assist efforts to increase vaccine uptake by farmers in Vietnam, thus reducing need for antibiotic therapy and consequent problems associated with bacterial antibiotic resistance. In turn, this will aid efforts to enhance the environmental sustainability of the striped catfish sector in Vietnam, which supports livelihoods and provides a nutritious animal protein source for people across the world.

## Data availability statement

The datasets presented in this study can be found in online repositories. The names of the repository/repositories and accession number(s) can be found in the article/Supplementary material.

## Ethics statement

The animal study was reviewed and approved by the University of Stirling Animal Welfare and Ethical Review Body.

## Author contributions

KB: conceptualisation, methodology, formal analysis, investigation, data curation, writing—original draft, writing—review and editing, visualization, and funding acquisition. TN: conceptualisation, investigation, resources, writing—review and editing, supervision, project administration, and funding acquisition. MB: conceptualisation, methodology, formal analysis, investigation, data curation, writing—original draft, writing—review and editing, and visualization. DH: investigation, resources, writing—review and editing, and funding acquisition. RH: conceptualisation and writing—review and editing. AA: conceptualisation, methodology, writing—review and editing, supervision, project administration, and funding acquisition. AD: conceptualisation, methodology, formal analysis, data curation, writing—original draft, writing—review and editing, visualization, supervision, project administration, and funding acquisition. All authors contributed to the article and approved the submitted version.
