# Supplementary material for: Aeromonas hydrophila ST251 and Aeromonas dhakensis are major emerging pathogens of striped catfish in Vietnam
Source: Front Microbiol. 2023 Jan 26;13:1067235. doi: 10.3389/fmicb.2022.1067235 (PMC9924233; doi:10.3389/fmicb.2022.1067235)
Supplement: Supplementary file 7 [file Data_Sheet_7.pdf]

Aeromonas hydrophila ST251 and *Aeromonas dhakensis* are major emerging pathogens of striped catfish in Vietnam

1 SUPPLEMENTARY TABLES

Table S1

Isolate details including host species, sample site, collection date and geographic origin. (CSV)

Table S2

Phenotyping and genotyping summary of isolates. NF, not found. (CSV)

Table S3

Primer sets of the MLST loci and *16S rRNA* gene, including expected amplicon sizes and references. (CSV)

Table S4

A. hydrophila and *A. dhakensis* genomes used in the comparative genomic analyses. For each isolate the assembly (or sample) accession, assigned bacterial species name, isolate/strain name, and source of the isolate including geographic origin and date of collection is provided. (CSV)

Table S5

Genome classification details, vAh status, average nucleotide identity (ANI), antibiotic-resistance genes and MLST results. (CSV)

Table S6

Diagnostic primer sets for distinguishing *A. dhakensis* and *A. hydrophila* vAh ST251 including sequences and optimal annealing temperatures. (CSV)

2 SUPPLEMENTARY FIGURES

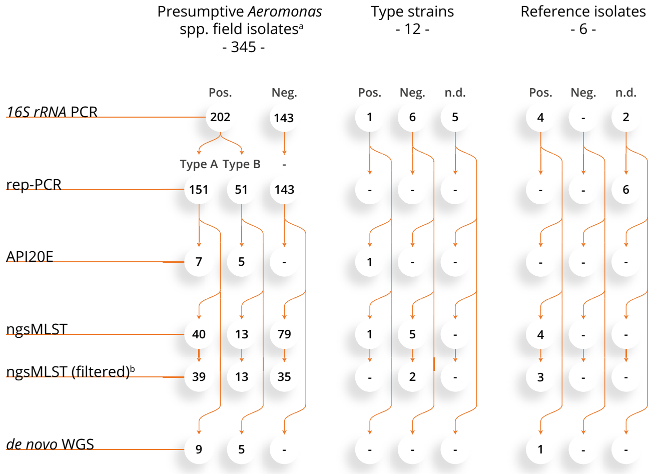

Figure S1. Summary of the analyses performed on isolates included in this present study. The 345 field isolates formed colonies on isolation media consistent with *Aeromonas* spp. and were purified. Samples had been derived from *P. hypophthalmus* individuals exhibiting classical signs of motile *Aeromonas* septicaemia. This study included 12 type strains of *Aeromonas* spp. and six reference isolates. This figure should be consulted with the details in Supplementary Tables S1 and S2. [a] Isolates recovered on *Aeromonas* selective media and purified. [b] Number of concatenated sequences meeting the threshold of average coverage above 400×. Sample details and genotyping results are provided in Supplementary Table S2. n.d., not done.

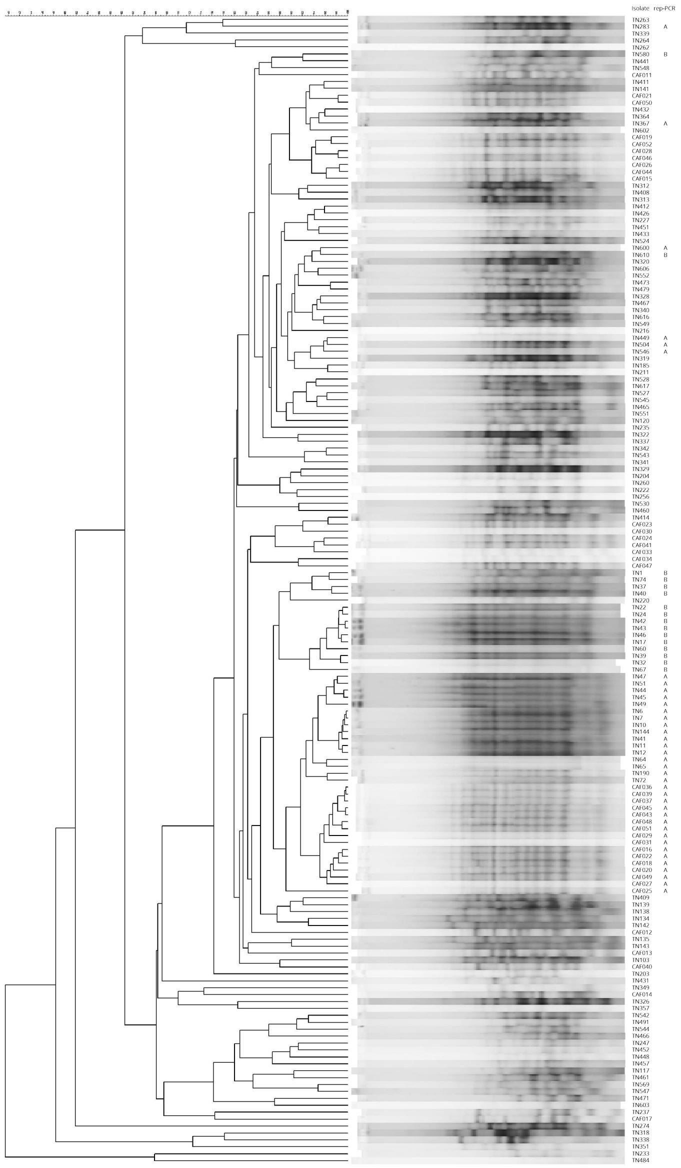

Figure S2. Dendrogram comparing the rep-PCR profiles of 166 representative isolates from *P. hypophthalmus* suspected to be *Aeromonas hydrophila* and *Aeromonas* spp. Dendrogram based on the UPGMA method and Pearson coefficient. Isolate name and the rep-PCR group is provided.

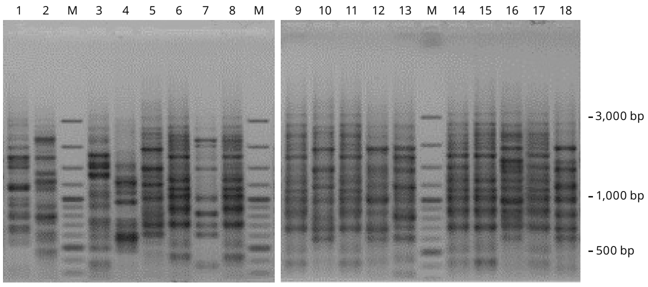

Lane	Isolate	Year	Province	rep-PCR type
1	CAF011	2015	Ben Tre	*Aeromonas* spp.
2	CAF012	2015	Ben Tre	*Aeromonas* spp.
M				
3	CAF013	2015	Tien Giang	*Aeromonas* spp.
4	CAF014	2015	Tien Giang	*Aeromonas* spp.
5	CAF015	2015	An Giang	*Aeromonas* spp.
6	CAF016	2015	An Giang	*A. hydrophila* A
7	CAF017	2015	Vinh Long	*Aeromonas* spp.
8	CAF018	2015	Vinh Long	*A. hydrophila* A
M				
9	CAF043	2018	Hậu Giang	*A. hydrophila* A
10	CAF044	2018	Hậu Giang	*Aeromonas* spp.
11	CAF045	2018	Dong Thap	*A. hydrophila* A
12	CAF046	2018	Dong Thap	*Aeromonas* spp.
13	CAF047	2018	Can Tho	*Aeromonas* spp.
M				
14	CAF048	2018	Can Tho	*A. hydrophila* A
15	CAF049	2018	Vinh Long	*A. hydrophila* A
16	CAF050	2018	Vinh Long	*Aeromonas* spp.
17	CAF051	2019	An Giang	*A. hydrophila* A
18	CAF052	2019	An Giang	*Aeromonas* spp.

Figure S3. Representative rep-PCR profiles obtained from *A. hydrophila* rep-PCR type A and *Aeromonas* spp. isolates (cropped gels). Lane M is the GeneRuler 100 bp Plus DNA Ladder (Thermo Fisher Scientific). Numbers on the right indicate the size of the bands of the molecular size marker in base pairs.

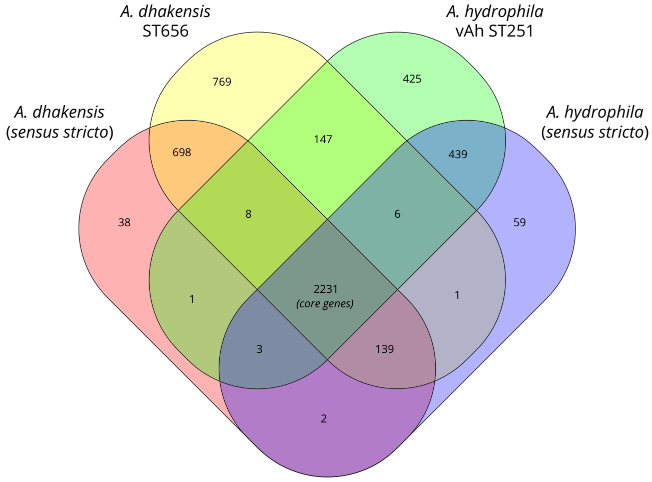

Figure S4. Four-way Venn diagram of the genes shared (95% presence in genomes) between strains and species. *Sensus stricto* denotes all the available genomes for the species, excluding the genomes of the outbreak sequence types (STs). Residual small numbers are the result of the limited number of genomes compared and the application of the 95% presence threshold.

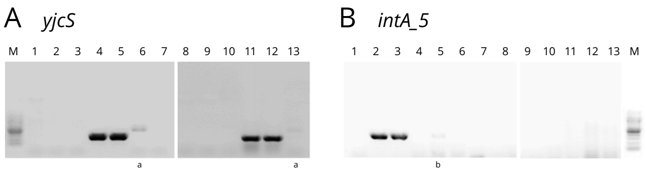

Lane	Isolate	Species	*yjcS* PCR	*intA_5* PCR
1	T4	*A. hydrophila*	-	-
2	TN27	vAh ST251	-	+
3	TN1	vAh ST251	-	+
4	TN5	*A. dhakensis*	+	-
5	TN49	*A. dhakensis*	+	-[b]
6	ATCC 7966^{T}	*A. hydrophila*	-[a]	-
7	NCIMB 13015^{T}	*A. veronii*	-	-
8	CECT 4227^{T}	*A. bestiarum*	-	-
9	B2/12	*A. hydrophila*	-	-
10	NCIMB 12065^{T}	*A. sobria*	-	-
11	CIP 107500^{T}	*A. dhakensis*	+	-
12	CECT 7289^{T}	*A. dhakensis*	+	-
13	NCIMB 13442^{T}	*A. encheleia*	-[a]	-

Figure S5. Summary diagnostic PCR screening for *A. dhakensis* and *A. hydrophila* vAh ST251. (**A**) *A. dhakensis* diagnostic *yjcS* PCR specificity check at 52°C. [a] At 62°C the non-specific products were not present. (**B**) *A. hydrophila* vAh ST251 diagnostic *intA_5* PCR specificity check at 52°C. [b] At 58°C the non-specific product was not present.
